# The Power of Heuristics in Predicting Fracture Nonunion

**DOI:** 10.3390/jcm14082713

**Published:** 2025-04-15

**Authors:** Jonas Armbruster, Eva Steinhausen, Simon Hackl, Marie K. Reumann, Dirk Stengel, Frank Niemeyer, Gregor Reiter, Paul Alfred Gruetzner, Holger Freischmidt

**Affiliations:** 1 BG Klinik Ludwigshafen, Department for Orthopaedics and Trauma Surgery, Heidelberg University, Ludwig-Guttmann-Str. 13, 67071 Ludwigshafen, Germany; 2 Department of Trauma, Hand and Reconstructive Surgery, University Hospital Essen, Hufelandstr. 55, 45147 Essen, Germany; 3 Department of Orthopedic and Trauma Surgery, BG Klinikum Duisburg, University of Duisburg-Essen, 47249 Duisburg, Germany; 4 Department of Trauma Surgery, BG Unfallklinik Murnau, Professor-Küntscher-Str. 8, 82418 Murnau, Germany; 5 Department for Trauma and Reconstructive Surgery, Eberhard Karls University Tuebingen, BG Klinik Tuebingen, Schnarrenbergstr. 95, 72076 Tuebingen, Germany; 6 BG Kliniken—Klinikverbund der Gesetzlichen Unfallversicherung gGmbH, Leipziger Pl. 1, 10117 Berlin, Germany; 7 OSORA Medical GmbH, Industriestr. 10, 89231 Neu-Ulm, Germany

**Keywords:** nonunion, heuristic reasoning, fracture healing, cognitive biases

## Abstract

**Background/Objectives:** Although extensive research on risk factors for nonunion development has been published, clinicians frequently rely on heuristic reasoning—intuitive, experience-based decision-making—to predict nonunions. However, the accuracy of these intuitive assessments and the influence of clinician experience remain uncertain. This study aims to assess clinicians’ diagnostic accuracy in predicting nonunion, investigate the impact of experience on predictive performance, and identify patient-specific factors contributing to diagnostic errors. **Methods:** This retrospective, multi-center cohort study included 98 patients with surgically treated tibial shaft fractures between 2018 and 2023 from four level-one trauma centers in Germany. Fracture outcomes were classified as either nonunion (*n* = 20) or regular fracture healing (*n* = 78). Patient cases were presented to 24 clinicians. Each clinician independently assessed preoperative and postoperative biplanar X-rays and patient histories to predict fracture healing. **Results:** Clinicians’ sensitivity significantly improved from 50.4% to 60.2%, while specificity declined (74.0% to 70.7%) with the addition of postoperative information. No significant differences in predictive performance were observed across different levels of clinician experience. Changes in assessment after reviewing postoperative information were equally likely to be beneficial or detrimental. Certain patient factors, including obesity and smoking, influenced prediction errors. **Conclusions:** This study is the first to assess heuristic reasoning in nonunion prediction. The findings suggest that clinician experience does not significantly enhance diagnostic accuracy under limited-information conditions. Patients should be informed that predicting individual nonunion risk remains challenging. Larger studies are needed to explore the role of patient-specific factors and refine clinical decision-making in fracture healing prognosis.

## 1. Introduction

Nonunions (NUs) are recognized as one of the most severe and challenging complications in orthopedics and traumatology [1]. An NU is defined as the failure of a fracture to consolidate without additional surgical intervention 6 to 9 months after the index event [2]. Treatment often requires multiple surgical procedures, the use of autologous or allogenic bone grafts to fill bone defects, and, in some serious cases, even amputation [3]. NUs are associated with prolonged pain and functional deficits, contributing to a considerable decline in quality of life comparable to advanced heart disease or stroke [4,5,6]. The socioeconomic burden of NUs is substantial, with costs far exceeding those of regular fracture healing [7,8].

NUs are commonly observed after fractures of the long bones, predominantly the tibia. Incidence rates of up to 30% have been reported, depending on risk factors such as smoking, diabetes mellitus, soft tissue damage, open wounds, and subsequent infection [7,9]. Certain fracture patterns significantly influence the risk of NU as well, with multi-fragmentary fractures (classified as type C in the AO/OTA system) exhibiting higher NU rates compared to simpler fractures (Type A or B) [9].

Optimal primary management of fractures is crucial in preventing NUs [10]. Given that infection is a major contributing factor, adherence to surgical asepsis and meticulous debridement of infected or necrotic tissue are essential [11]. Additionally, addressing patient-related variables associated with impaired bone metabolism further reduces the risk of NU [12,13]. From a surgical perspective, fracture gaps must be minimized to facilitate direct bone healing, and implants providing proper construct stability are critical. While too-rigid interfaces may hinder micromovements essential for fracture healing, insufficient firmness can result in excessive shear at the fracture site, predisposing patients to NU [14].

As these influencing factors are well documented in the literature, highlighted by a recent meta-analysis encompassing 111 studies and over 40,000 patients [9], clinicians often intuitively understand whether a fracture will heal or result in an NU. In scientific terms, this intuition is named heuristic reasoning. In psychology, heuristics are mental shortcuts that enable individuals to make decisions based on limited information [15]. While heuristic approaches may overlook certain details, they can often yield highly accurate results [15,16]. This is particularly valuable if experienced clinicians are consulted, whereas caution is necessary among younger clinicians or medical students [16,17,18]. Furthermore, clinicians of different clinical backgrounds may assign varying levels of importance to relevant information, leading to divergent heuristic decisions based on the same data [19]. Regarding experience, one study found no evidence of age-related differences in the use of common heuristics. However, it did reveal that younger adults were more susceptible to the sunk-cost bias—the tendency to continue an endeavor due to prior investments of time, effort, or money, even when the costs outweigh the benefits—suggesting a greater likelihood of persisting in suboptimal decisions compared to older adults [20].

Little is known about the role of heuristics in predicting fracture outcomes or diagnosing NUs. It remains unclear whether more experienced surgeons achieve greater diagnostic accuracy when relying on limited information. Additionally, questions persist regarding whether clinically known patient factors contribute to diagnostic bias and in which way. This study aims to address these gaps in the literature.

## 2. Materials and Methods

The Ethics Committee of the Medical Association of Rhineland-Palatinate, Germany, approved this study (approval number: 2023-17066-retrospektiv).

This multicenter, retrospective cohort study was conducted at four German level-one trauma centers and included anonymized data from patients treated for tibial shaft fractures between January 2018 and December 2023. The inclusion and exclusion criteria are outlined in Table 1.

**Figure 1:**
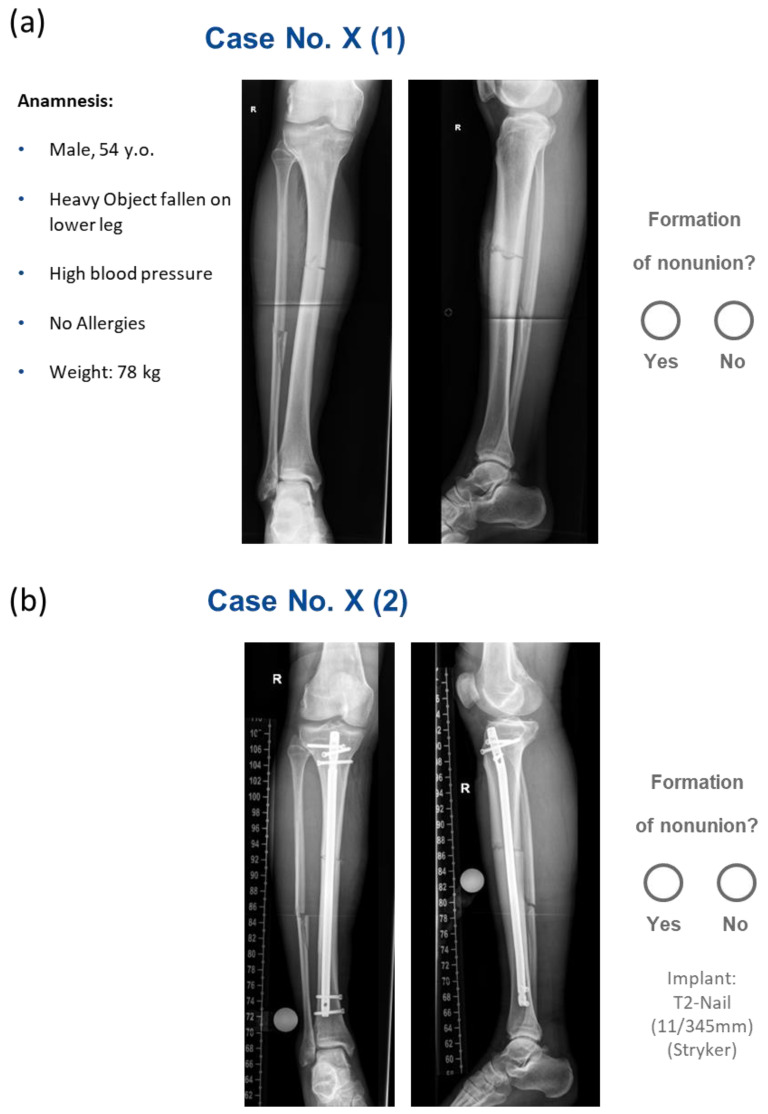
Demonstration of a patient case as presented to the clinical raters for healing prediction: (**a**) preoperative biplanar X-rays and patient background information; (**b**) postoperative biplanar X-rays (original text in German). R = right side.

We enrolled 98 patients, 20 of whom were diagnosed with NU, while 78 subjects showed normal fracture healing. Figure 1 illustrates a representative case.

Relevant demographic variables and medical history, including obesity, hypertension, and smoking, were documented and displayed alongside pre- and postoperative biplanar X-rays to the participating clinicians. Table 2 provides a summary of patient information.

According to the definition outlined in the Introduction Section (Section 1), fractures that did not progress to healing within a timeframe of 6 to 9 months after initial surgical treatment were considered NUs.

A total of twenty-four raters (six from each hospital) were recruited, representing a diverse range of experience and professional roles. These included the head of the surgical department, a consultant, a specialist, a resident, and an intern, all with backgrounds in orthopedic or trauma surgery. Additionally, one radiologist from each site was included, given their expertise in interpreting biplanar radiographs. Each rater independently evaluated each case to determine whether the fracture would heal or progress to an NU. This assessment was made in two stages. Initially, preoperative information and X-ray images obtained at the time of injury were presented, and clinicians were asked to predict the outcome. Subsequently, postoperative radiological results were provided alongside surgical information, and raters then reassessed the outcomes.

Statistical analysis: Data were collected using Microsoft Excel (Microsoft Corporation, Redmond, WA, USA) and analyzed using JASP v0.19.1 [22], pandas v2.2.3 [23], and seaborn v0.13.2 [24]. Fracture classification adhered to the Arbeitsgemeinschaft für Osteosynthesefragen (AO; Association for the Study of Internal Fixation) and the Orthopaedic Trauma Association (OTA). To quantify the predictive power of the experience-based risk assessment conducted by the clinical raters, a binary confusion matrix was computed for each rater, both for preoperative and postoperative assessments. A fracture union outcome y or prediction $\hat{y}$ was coded as 0 (“negative”), while an NU was coded as 1 (“positive”). False positives (FPs) were cases in which raters incorrectly predicted an NU. In contrast, false negatives (FNs) represented fractures expected to heal resulting in NUs. Based on these confusion matrices, the binary classification metrics—sensitivity, specificity, positive predictive value (PPV), and negative predictive value (NPV)—were calculated to identify the factors most frequently leading to incorrect predictions; the prediction error ε was defined as $\varepsilon =\hat{y}-y$, where false positives (FP) corresponded to an error value of 1, and false negatives (FN) correspond to an error value of −1. A multiple linear regression model was applied to estimate the association between patient characteristics and ε.

To test the statistical significance of differences between *n* > 2 parametric variables, a one-way analysis of variance (ANOVA) followed by Bonferroni’s multiple comparisons test was performed. Two parametric variables, like the same group’s preoperative and postoperative performance metrics, were compared using Student’s paired *t*-test. Equal variances were assumed, depending on Levene’s test. A two-sided *p*-value < 0.05 was considered to show statistical significance, i.e., indicating that the results were not explainable by chance alone.

## 3. Results

### 3.1. Patient Characteristics

**Figure 2:**
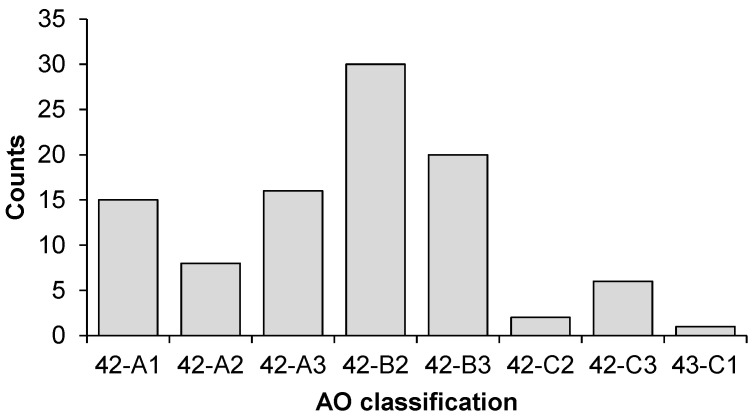
Fracture types according to the AO classification.

Ninety-eight patient cases were included in this study. Table 3 summarizes their main characteristics. Most tibial shaft fractures were classified as 42-B (*n* = 50), followed by 42-A (*n* = 39) and 42-C (*n* = 9). Upon further review, one patient was reclassified as having a distal tibia fracture (AO 43), as an intra-articular fissure was not identified during the initial assessment. Figure 2 shows the distribution of fracture types according to the AO classification.

### 3.2. Rater Characteristics

A total of 24 raters participated in this study. As expected, higher professional positions were associated with more years of experience. Table 4 provides a detailed summary of the characteristics of the raters.

### 3.3. Clinicians’ Performance Metrics in Predicting Nonunion Development

**Figure 3:**
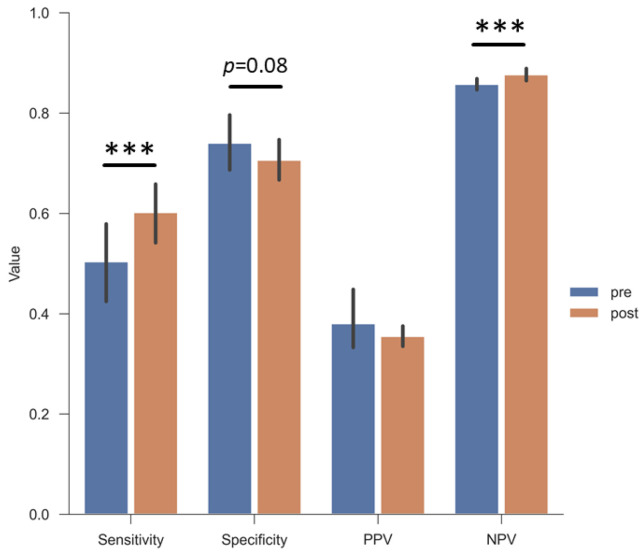
Performance ratings of all raters combined: Postoperative information like surgical reduction and implant information significantly increases the sensitivity and reduces clinician raters’ specificity. Sensitivity_preop_ = 50.4% vs. Sensitivity_postop_ = 60.2%, *p* < 0.001; Specificity_preop_ = 74.0% vs. Specificity_postop_ = 70.7%, *p* = 0.08; PPV_preop_ = 38.1% vs. PPV_postop_ = 35.5%, *p* = 0.40; NPV_preop_ = 85.8% vs. NPV_postop_ = 87.7%, *p* < 0.001. *** = *p* < 0.001. Error bars: bootstrapped 95% confidence intervals.

While sensitivity increased significantly with the addition of postoperative information from 50.4 to 60.2%, specificity decreased from 74.0% to 70.7% (*p* = 0.08). The positive predictive value (PPV) decreased marginally between preoperative and postoperative evaluations, whereas the negative predictive value (NPV) increased slightly (84.3% to 86.3%) when postoperative information was incorporated. Figure 3 presents the results.

### 3.4. No Significant Difference in Prediction Metrics Across Clinician Roles

**Figure 4:**
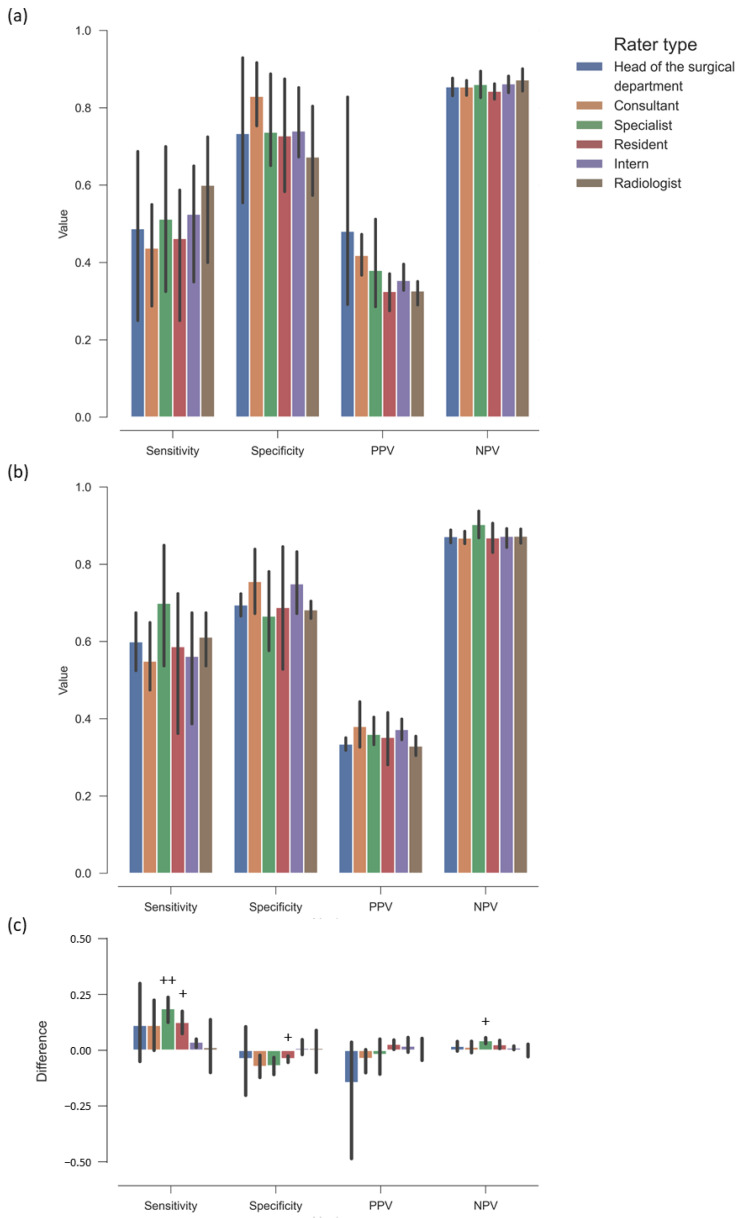
Performance ratings of raters categorized by professional position: (**a**) Preoperative evaluation. No significant differences were observed between positions. (**b**) Postoperative evaluation. No significant differences were observed between positions. (**c**) Delta between preoperative and postoperative evaluation. Postoperative information, such as surgical reduction and implant details, led to a significant increase in sensitivity, particularly among specialists and residents, but also reduced their specificity. Additionally, the NPV of specialists showed a significant but marginal increase. However, no significant differences were observed in the delta between preoperative and postoperative evaluations across the categorized positions. ++ = *p* < 0.01, + = *p* < 0.05, preoperative to postoperative metric. Error bars: bootstrapped 95% confidence intervals.

To assess whether a clinician’s position influences predictive performance, the results were categorized based on the clinician’s stage of training. There were no significant differences in sensitivity, specificity, PPV, or NPV across different experience levels. Figure 4 presents the results.

### 3.5. Greater Experience Does Not Correlate with Improved Intuition

**Figure 5:**
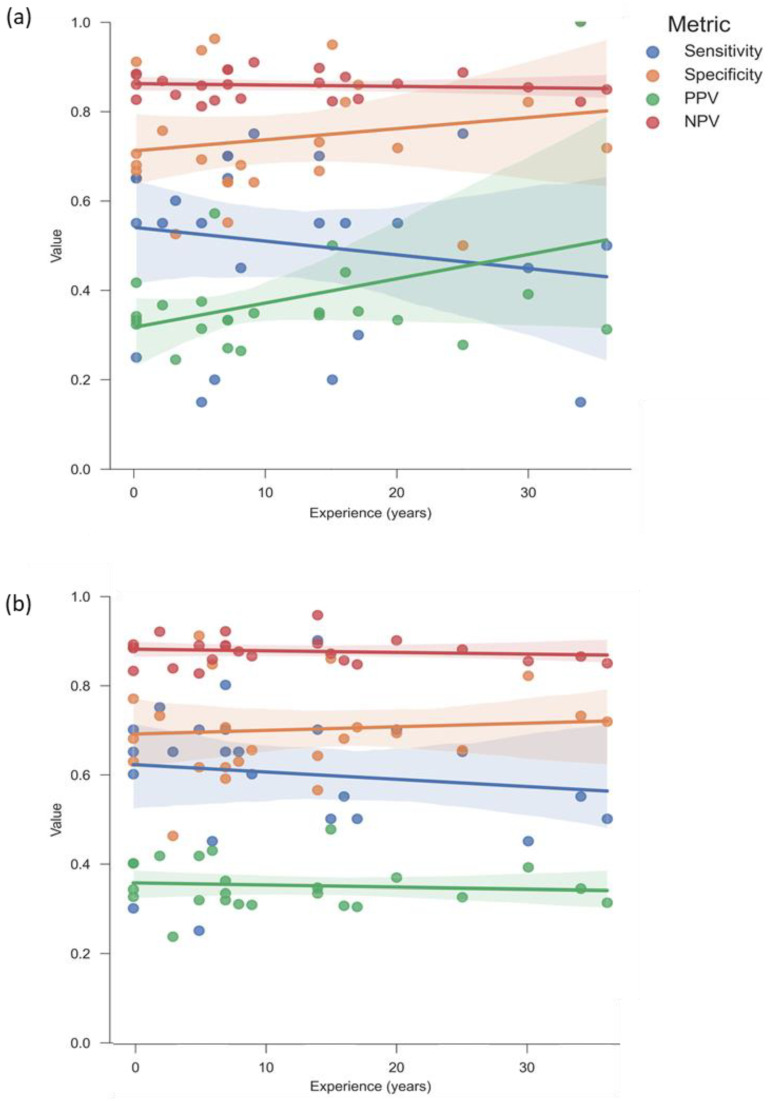
Correlation between raters’ performance metrics and experience. No significant correlation was found between performance metrics and rater experience. Preoperative PPV showed the highest correlation with experience but remained insignificant (Pearson’s *r* = 0.386, *p* = 0.062): (**a**) preoperative evaluation; (**b**) postoperative evaluation. Shaded areas indicate 95% confidence intervals.

To further examine the impact of experience on clinicians’ ratings, performance metrics were analyzed in relation to years of clinical experience. PPV tended to correlate with experience, but only in the preoperative evaluation. No significant correlation was observed. Figure 5 presents the results.

### 3.6. Changing Initial Ratings After Receiving Postoperative Information Did Not Improve Clinicians’ Accuracy

**Figure 6:**
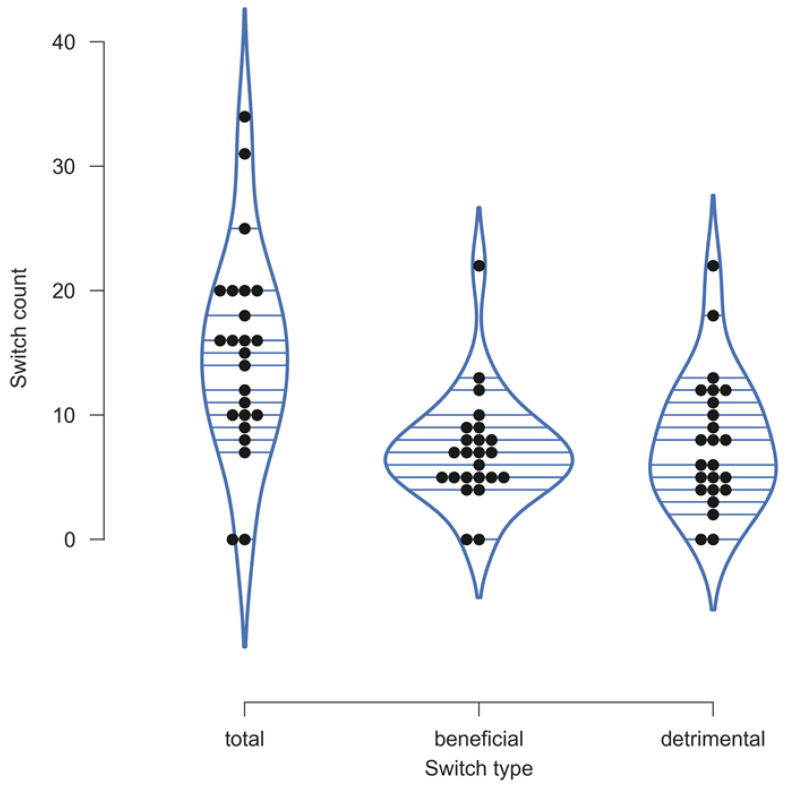
Violin plots illustrating changes from preoperative to postoperative ratings after receiving additional information. The plots display the total number of changes and the counts of beneficial and detrimental changes for each individual clinician (black dots).

When clinicians revised their evaluations after receiving additional postoperative information, these changes were equally likely to be beneficial or detrimental. Figure 6 presents the results.

### 3.7. Clinically Relevant Patient Characteristics Influence Prediction Error of Postoperative Evaluation

**Figure 7:**
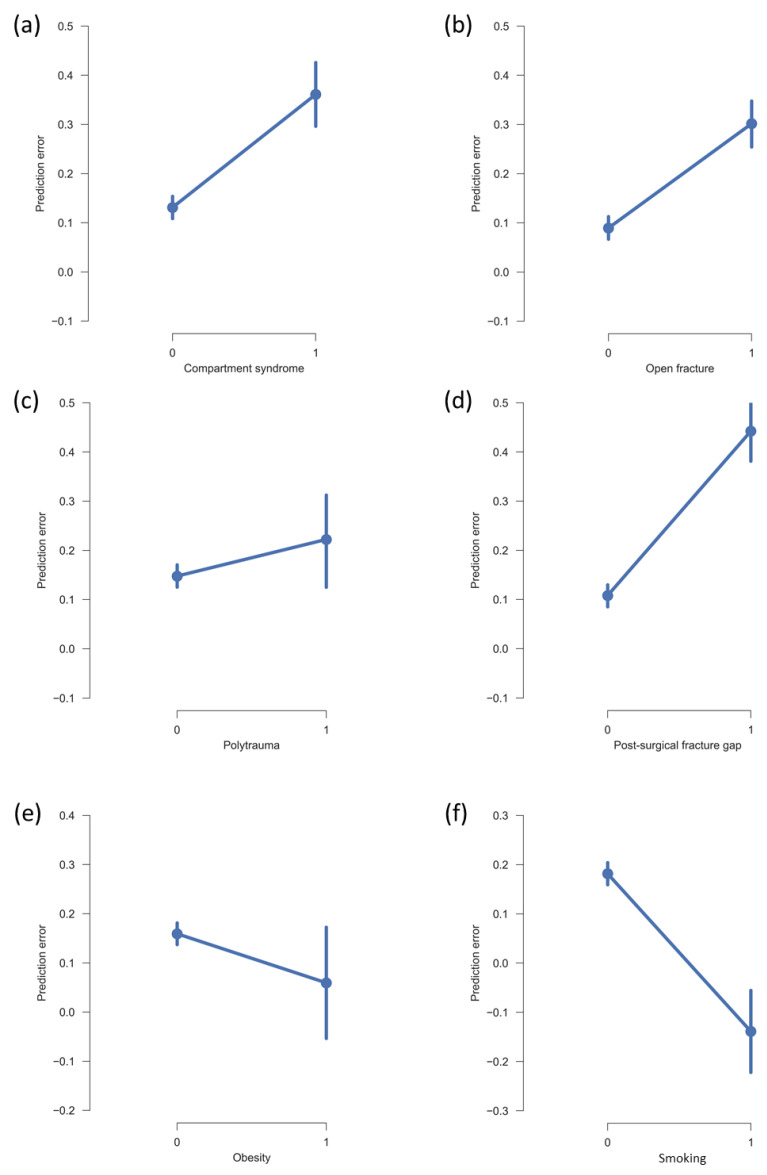
Mean prediction error by clinicians, stratified by the presence (1) or absence (0) of clinically relevant patient factors. Positive prediction errors reflect a tendency to overestimate the factor’s impact (false positives), whereas negative prediction errors indicate underestimation (false negatives): (**a**) compartment syndrome; (**b**) open fracture; (**c**) polytrauma; (**d**) post-surgical fracture gap (>4 mm); (**e**) obesity; (**f**) smoking. Error bars: bootstrapped 95% confidence intervals. Note the adjusted y-axis scales in (**e**,**f**) to better fit the data.

Patient characteristics—including obesity, smoking, and a postoperative fracture gap greater than 4 mm—were evaluated during the processing of case vignettes. Clinicians noticeably overestimated the risk of NU in cases of compartment syndrome, open fractures, and a postoperative fracture gap >4 mm. In contrast, polytrauma and obesity did not affect the prediction error, while the impact of smoking was underestimated. Due to the small case numbers for some of these factors (see Table 3), individual effects were not statistically analyzed. Figure 7 displays the mean prediction error of clinicians when these characteristics were present versus absent.

## 4. Discussion

### 4.1. Principal Findings

To our knowledge, this is the first study to investigate the role of heuristics in predicting NU after tibia fractures. This research project analyzed a cohort of 98 case vignettes, in which 20 tibial fractures resulted in an NU (20.4%), while 78 (79.6%) cases progressed with physiological fracture healing (see Table 3). This distribution aligns with reported NU rates in the literature, which range from 0% to 42.7% [9]. The cohort investigated is representative, comprising a predominantly male population (69%) with a mean age of 40 years [25]. Various risk factors were present, including open fractures (30%), obesity (7%), and smoking (9%). Additionally, compartment syndrome was diagnosed in 9% of cases, closely matching the reported incidence of 8% in the literature [26]. Soft tissue damage was documented in 29% of cases, which is somewhat lower than the rate suggested in the literature [27].

The overall sensitivity of clinician raters in excluding the progress to NUs was 50% when only preoperative information was provided. It improved to 60% with the inclusion of additional postoperative data (see Figure 3). The specificity declined from 74% to 70%. No significant differences were observed when performance metrics were analyzed according to clinician roles or correlated with years of experience (see Figure 4a,b and Figure 5a,b). Sensitivity significantly increased between preoperative and postoperative evaluations amongst specialists and residents. However, specificity significantly decreased in specialists. This trend was not observed among consultants and heads of surgical departments, suggesting that these groups were less likely to overestimate the risk of NU after reviewing postoperative information (see Figure 4c).

An analysis of changes between preoperative and postoperative evaluations did not reveal a consistent pattern. In some cases, postoperative information improved accuracy; in others, it had a contradicting effect (see Figure 6). Furthermore, the present data provided no evidence of a sunk cost fallacy, as reported by Taylor et al. [20], among clinician raters when diagnosing NU. Clinicians with more years of experience did not differ significantly in their total, beneficial, or detrimental switch counts compared to those with less experience (data not shown).

### 4.2. Prediction Errors of Clinician Raters Are Associated with Clinically Relevant Patient Factors

Patient-, injury-, and management-related features have been recognized as key determinants of post-surgical fracture outcomes [7,9,28,29]. Tian et al. systematically reviewed these variables in a meta-analysis encompassing over 40,000 patients [9]. They identified 15 items significantly influencing the likelihood of fracture union: age > 60 years, male gender, smoking, body mass index > 40, diabetes mellitus, intake of nonsteroidal anti-inflammatory drugs (NSAIDs) and/or opioids, fractures of the middle and distal tibia, high-energy and/or open fractures, Gustilo–Anderson grade IIIB or IIIC soft tissue injury, AO-type C fractures, open reduction, and surgical site infection. This spectrum compares well to that previously shown by Zura et al. in a nationwide U.S. claims database of approximately 90.1 million participants [25]. Due to the limited sample size, we were unable to investigate the contribution of all factors to clinical prediction thoroughly (see Table 3).

Clinicians overestimated the risk of NU if there was compartment syndrome, an open fracture, or a postoperative fracture gap > 4 mm. Although previous studies have demonstrated that these factors significantly affect fracture healing [9,25], their influence in the present cohort was less pronounced than anticipated by clinicians. Polytrauma and obesity did not affect accuracy, while the impact of smoking was underestimated. These findings highlight the challenges of accurately assessing the influence of individual patient factors on fracture healing outcomes. A potential solution may be to train machine learning algorithms using this and future larger datasets, as such algorithms are well suited for recognizing recurring patterns. Further studies are required to assess the performance of machine learning models in this context.

### 4.3. Heuristic Biases of Tibial Nonunion Diagnosis in Context

Cognitive bias can negatively affect surgical performance and patient outcomes at any stage of care [30]. Edelstein et al. showed that heuristic-based decision-making may lead to erroneous assumptions and suboptimal choices between total hip arthroplasty and hemiarthroplasty in patients with femoral neck fractures [31]. Similarly, Janssen et al. demonstrated that orthopedic surgeons are prone to common cognitive biases, including confirmation bias and anchoring [32].

The present study revealed some confirmation bias where participants tend to favor or recall information that aligns with their existing beliefs while discounting contradictory evidence. Some clinicians did not revise their evaluations at all after reviewing the postoperative information, while others made only minor adjustments. This pattern suggests that additional data were sometimes underutilized, despite their potential influence on the likelihood of nonunion. Notably, the total number of evaluation changes did not correlate with clinical experience or professional role, suggesting that more experienced physicians may just be as vulnerable to confirmation bias as their less experienced colleagues.

Anchoring describes a phenomenon in which individuals rely too heavily on the first piece of information (the “anchor”) when making subsequent decisions, even when new or more relevant data become available. Failing to adjust an assessment after reviewing postoperative findings may reflect anchoring bias, confirmation bias, or both. In practice, these two biases exhibit similar tendencies—a reluctance to update beliefs based on new information—so a lack of “postoperative switches” might be driven by overlapping cognitive factors.

A potential solution to suboptimal heuristic reasoning is to use objective scoring systems. Such tools can support clinical decision-making in orthopedics, traumatology, and other specialties; however, they have also been subject to scrutiny [33,34,35]. Regarding orthopedic research, recent work evaluating three recognized nonunion scores (FRACTING, NURD, LEG-NUI) in patients with tibial shaft fractures demonstrated high specificity (86.5–96.6%) but low sensitivity (14.6–63.4%) in predicting NU [36]. Compared with the present findings, these scoring methods could improve specificity for diagnosing nonunion but are unlikely to increase sensitivity, which is essential for identifying high-risk patients at an early stage. Consequently, some authors propose integrating artificial intelligence into clinical decision-making [37], which could represent a promising avenue for refining the diagnostic process in nonunion detection, as mentioned above.

### 4.4. Limitations

This study has several limitations. First, clinician raters could not access all the information typically available in a hospital setting. For example, while CT scans often provide additional details about fracture patterns, clinicians in this study were limited to evaluating X-ray images. Additionally, soft tissue injuries may have been underreported, as indicated by the discrepancy between detected injuries (see Table 3) and those reported in the literature (see Section 4.1). As clinicians know that most fractures have some degree of surrounding soft tissue injury, clinicians are likely to include this knowledge into their evaluation regardless of whether low-grade soft tissue injuries are reported or not.

Furthermore, this study included only cases from level 1 trauma centers in a developed country, where surgical process quality and outcomes are expected to be uniformly high. This uniformity made distinguishing between “good” and “poor” surgical treatments challenging, potentially leading to an underestimation of clinician heuristics.

Postoperative evaluation was based solely on radiographic imaging and implant information, but no data on postoperative weight-bearing were provided. Moreover, data on postoperative infections—an important factor known to impact patient outcomes—were not included. However, since this study aimed to investigate heuristics based on perioperative information, this limitation was an inherent aspect of the study design.

Finally, the number of patients in certain subgroups was too small to draw definitive statistical conclusions regarding the effect of specific patient factors. Larger studies are needed to investigate these associations.

## 5. Conclusions

This study represents the first analysis of clinician heuristics in predicting NUs, highlighting the inherent complexity of forecasting fracture outcomes. Notably, within this cohort and under limited information, neither clinician position nor experience significantly influenced diagnostic metrics. Clinical assessment of the risk of NU following fractures remains challenging. Future research should explore the potential of machine learning algorithms for early NU risk estimation. Furthermore, larger studies are needed to validate these findings and assess the specific impact of patient factors through subgroup analyses.

## Figures and Tables

**Table 1:** Inclusion and exclusion criteria for patient selection.

Inclusion	Exclusion
Tibial shaft fractures classified as AO Type 42, including all morphological subcategories Surgical management (plating or intramedullary nailing) performed between January 2018 and December 2023	Insufficient follow-up (less than 9 months) or until osseous consolidation
	No biplanar radiographs taken at the date of injury and immediately postoperatively available

**Table 2:** Patient information in addition to pre- and postoperative biplanar X-rays.

Parameter Group	Parameter [Unit]
Preoperative	Age [years] Sex [male/female] Weight [kg] Past medical history Compartment syndrome [yes/no] Soft tissue damage, according to Tscherne und Oestern [21]: ○ Closed fractures [Grade I–III] ○ Open fractures [Grade I–IV] One-step or two-step surgical protocol Polytrauma Previous infection [yes/no] Plastic soft tissue coverage [yes/no] Use of additional allograft or autograft Allergies
Postoperative	Name and size of orthopedic implant

**Table 3:** Patient characteristics. Percentage rounded to whole numbers. n/a = not assessable; TEN = Titanium Elastic Nail.

Patient Characteristics		Frequency	Percent [%]	Mean ± SD
Age [years]	Total	98	100	39.9 ± 16.5
	Male	68	69	39.8 ± 16.7
	Female	30	31	40.1 ± 16.0
Treatment	Nail	70	71	
	Plate	27	28	
	TEN	1	1	
Open fracture	Yes	29	30	
	No	69	70	
Obesity	Yes	7	7	
	No	91	93	
Smoking	Yes	9	9	
	No	89	91	
Compartment syndrome	Yes	9	9	
	No	89	91	
Polytrauma	Yes	6	6	
	No	92	94	
Plastic soft tissue coverage	Yes	3	3	
	no	95	97	
Fibula fractured	Yes	76	78	
	No	22	22	
Soft tissue damage	0° or n/a	69	70	
	1°	18	18	
	2°	7	7	
	3°	4	4	

**Table 4:** Rater characteristics.

Position	Frequency [*n*]	Mean Experience ± SD [Years]
Heads of Surgical Departments	4	28.8 ± 6.5
Consultants	4	18.8 ± 6.5
Specialists	4	8.8 ± 3.1
Residents	4	3.8 ± 1.3
Interns	4	0.0 ± 0.0
Radiologists	4	10.0 ± 4.1

## Data Availability

The data presented in this study are available on request from the corresponding author due to data privacy and ethical restrictions.

## Abbreviations

AO	Arbeitsgemeinschaft für Osteosynthesefragen (Association for the Study of Internal Fixation)
BMI	Body mass index
CT	Computed Tomography
FN	False negative
FP	False positive
LEG-NUI	Lower Extremity Grading of Nonunion Index
NSAID	Nonsteroidal anti-inflammatory drug
NU	Nonunion
NURD	Nonunion Risk Determination Score
NPV	Negative predictive value
ORIF	Open Reduction and Internal Fixation
OTA	Orthopaedic Trauma Association
PPV	Positive predictive value
TEN	Titanium Elastic Nail
